# 以反复出血起病的意义未明的单克隆丙种球蛋白血症1例

**DOI:** 10.3760/cma.j.issn.0253-2727.2022.12.014

**Published:** 2022-12

**Authors:** 樱樱 肖, 颖 张, 春 曹, 云 罗, 世锋 娄

**Affiliations:** 重庆医科大学附属第二医院血液科，重庆 400000 Department of Hematology, the Second Affiliated Hospital of Chongqing Medical University, Chongqing 400000

患者，女，65岁，2021年6月10日因“左上肢瘀斑9个月余，左侧胸壁瘀斑7个月余”第一次入院。患者9个月前无明显诱因出现左上肢瘀斑，伴局部隐痛，当地医院查血常规无异常，患者未行进一步治疗。7个月前患者左侧胸壁出现类似瘀斑，未行特殊治疗。4个月前患者无明显诱因出现齿龈出血，就诊于外院，血浆凝血酶原时间（PT）>180 s，凝血酶时间（TT）32.50 s，血浆纤维蛋白原（FIB）0.30 g/L，D-二聚体32.07 mg/L，输注血浆、止血药物后好转出院。现患者为求进一步诊治入住我科，入院查体：左上肢可见1个15 cm×15 cm的瘀斑，左侧胸壁可见1个15 cm×10 cm瘀斑，其余未见异常。血常规示：RBC 4.53×10^12^/L，HGB 133 g/L，WBC 9.15×10^9^/L，中性粒细胞百分比76.5％，PLT 105×10^9^/L。肝肾功能正常。凝血功能示：凝血酶原活动度42％，PT 21.80 s，APTT 49.90 s，FIB<0.60 g/L，TT 32.40 s，纤维蛋白原降解产物>150 µg/ml，血浆D-二聚体>10 000 ng/ml，血管性血友病因子抗原百分比290％。血电解质示钙2.35 mmol/L，钾3.54 mmol/L，磷0.91 mmol/L，氯101.70 mmol/L。尿液及大便隐血均阴性。查凝血因子示活性均轻度减低。抗磷脂抗体谱正常，抗增殖细胞抗核抗体（+），其余自身抗体阴性。易栓症筛查示蛋白C活性76％，蛋白S活性10％。全脊柱核磁共振示颈、胸、腰椎退行性改变、腰椎间盘变性并L5/S1轻度膨出、腰骶部软组织水肿。骨盆X线示双侧髋关节退行性变。血管超声检查示三尖瓣中度关闭不全，左室舒张功能减低。四肢血管超声示右侧腘静脉附壁血栓形成。骨髓细胞检查示粒系成熟加速，浆细胞增多，骨髓增生活跃。骨髓流式细胞术分析示异常浆细胞占0.77％。免疫固定电泳可见单克隆IgG及λ轻链条带。免疫球蛋白测定：IgG 18.40 g/L、λ轻链17.10 g/L、M蛋白带可疑（β区带）。骨髓细胞FISH检测分析未见异常。腹壁皮肤活检刚果红染色阴性。出凝血相关基因高通量测序（NGS）检测：1号染色体109510247外显子发生杂合变异。入院后根据相关检查考虑诊断：意义未明的单克隆丙种球蛋白血症（MGUS）；凝血功能障碍；下肢深静脉血栓形成；皮肤软组织感染；皮下血肿；颈椎退行性病变；三尖瓣关闭不全。

入院后根据检查结果予维生素K1、氨甲环酸、注射用血凝酶止血，抑酸护胃，间断输注纤维蛋白原、新鲜冰冻血浆等。静脉彩超提示腘静脉血栓形成后，加用低分子肝素抗凝。患者左上肢瘀斑、疼痛、发黑，加用头孢唑林抗感染，局部换药。患者疼痛、水肿减轻且无新瘀斑形成后停用低分子肝素，改用利伐沙班抗凝治疗。复查凝血功能好转，考虑患者MGUS引起的临床并发症，予硼替佐米（1.35 mg/m^2^）联合地塞米松（40 mg）化疗1次后出院。院外继续每周1次硼替佐米化疗共8次（剂量同前），口服抗凝药物，患者未再出现出血、血肿、血栓等异常。患者3个月后入院复查，易栓症筛查示蛋白S活性33％。骨髓细胞学检查示骨髓增生活跃，骨髓流式细胞术示异常浆细胞占细胞总数的0.03％。根据检查结果予达雷妥尤单抗400 mg静脉输注清除残留浆细胞，院外口服来那度胺10 mg，每晚1次，口服两周后停药两周，利伐沙班10 mg每日1次维持治疗，定期每周复查血常规和凝血功能，未见异常。患者6个月后入院复查，凝血功能及免疫球蛋白正常，继续予达雷妥尤单抗巩固治疗，院外继续口服上述药物，用法如前，现患者继续随访中，未诉不适。

